# Three new species of *Begonia* (Begoniaceae) from Bahia, Brazil

**DOI:** 10.3897/phytokeys.44.7993

**Published:** 2015-01-13

**Authors:** Bernarda de Souza Gregório, Jorge Antonio Silva Costa, Alessandro Rapini

**Affiliations:** 1 Departamento de Ciências Biológicas, Universidade Estadual de Feira de Santana, Av. Transnordestina, s/n, Novo Horizonte, 44036-900, Feira de Santana, Bahia, Brazil; 2 Centro de Formação em Ciências Ambientais, Instituto Sosígenes Costa de Humanidades, Artes e Ciências (IHAC), Universidade Federal do Sul da Bahia (UFSB), BR 367, Km 10 da Rodovia Porto Seguro-Eunápolis - Centro de Convenções, 45.810-000, Porto Seguro, Bahia, Brazil

**Keywords:** Atlantic forest, Piedmont of Paraguaçu, Recôncavo, South coast, taxonomy, Mata Atlântica, Piemonte do Paraguaçu, Recôncavo, Litoral Sul, taxonomia

## Abstract

The taxonomic treatment of Begoniaceae for the state of Bahia, Brazil, led to the recognition of three new species of *Begonia* with narrow distributions, which are described and illustrated here: *Begonia delicata* Gregório & J.A.S. Costa, **sp. nov.** is a herb restricted to the region of the Recôncavo; *Begonia elianeae* Gregório & J.A.S. Costa, **sp. nov.** is a shrub endemic to the Atlantic forest of the southern part of the state; and *Begonia paganuccii* Gregório & J.A.S. Costa, **sp. nov.** is a subshrub known only from the type material, collected in the Piedmont of Paraguaçu. Notes on morphology, comparisons with morphologically similar species, etymology, geographic distribution, habitat and phenological data for each species are also presented. Furthermore, keys are provided as an aid to separating the new species from congeneric species that occur in their surroundings. Due to the sparse knowledge of the new species, there is as yet insufficient data to accurately assess their conservation status.

## Abstract (pt)

O tratamento taxonômico das Begoniaceae do estado da Bahia, Brasil, resultou no reconhecimento de três espécies novas de *Begonia* com distribuição restrita, as quais são descritas e ilustradas aqui: *Begonia delicata* Gregório & J.A.S. Costa, **sp. nov.** é uma erva restrita ao Recôncavo; *Begonia elianeae* Gregório & J.A.S. Costa, **sp. nov.** é um arbusto endêmico da Mata Atlântica do sul do estado; e *Begonia paganuccii* Gregório & J.A.S. Costa, **sp. nov.** é um subarbusto, conhecido apenas pelo espécime-tipo, coletado no Piemonte do Paraguaçu. São apresentados comentários morfológicos, comparações com espécies semelhantes, etimologia, distribuição geográfica, hábitat e dados fenológicos para cada espécie. Além disso, são fornecidas chaves de identificação para separá-las de espécies congenéricas que ocorrem na circunvizinhança dessas espécies. Devido ao conhecimento esparso das novas espécies, os dados ainda são insuficientes para classificá-las acuradamente quanto ao estado de conservação.

## Introduction

*Begonia* L. is one of the largest genera of Angiosperms (~1,500 species), known worldwide as ornamentals, with numerous hybrids and cultivars popular in the horticultural market (Neale et al. 2006). The genus probably arose in Africa but is most diverse in the Americas and Asia (Goodall-Coopestake et al. 2010), occurring in a variety of habitats, but mainly in moist and shady forests (Clement et al. 2004). Taxonomically, it is arranged in more than 60 sections (Doorenbos et al. 1998). Nevertheless, these sections are not morphologically consistent and diagnostic features of one section are often found in members of other sections (Forrest et al. 2005). Although phylogenetic studies in *Begonia* have been based on low density, world-wide sampling (e.g., Forrest et al. 2005) or focused only on species of certain Old World regions (e.g., Plana 2003; Thomas et al. 2011), several sections of *Begonia* were already shown to be poly- or paraphyletic, and the sectional circumscription of Neotropical groups appears to be highly problematic (Dewitte et al. 2011).

In the course of preparing the taxonomic treatment of *Begonia* for the state of Bahia, Brazil (Gregório 2014), in addition to field work in different habitats, specimens from 24 Brazilian herbaria—ALCB, BAH, BHCB, BRBA, CEN, CEPEC, HB, HEPH, HRB, HST (Herbário Sérgio Tavares), HUEFS, IBGE, IPA, MBM, MBML, PEUFR, R, RB, RBR, SP, SPF, UB, UFP and UPCB (acronyms according to Thiers et al. 2014)—and photos of specimens from seven herbaria from other countries (B, G, K, M, NY, P and US) were examined. This inventory recorded 37 species of *Begonia* for the state and recognised ten new synonyms in the genus. More than 80% of these species occur in Atlantic forest, 14 are endemic to Bahia and according to Jacques et al. (2013) six are endangered. In addition to *Begonia obdeltata* Gregório & E.L. Jacques, which also occurs in the state of Pernambuco (Gregório et al. 2014), three undescribed species of *Begonia* were discovered during the inventory. They have a narrow distribution and are described and illustrated here: *Begonia delicata* Gregório & J.A.S. Costa, endemic to the Recôncavo; *Begonia elianeae* Gregório & J.A.S. Costa, endemic to southern Bahia, and *Begonia paganuccii* Gregório & J.A.S. Costa, known only from the type specimen, collected in seasonal forest in the region of the Piedmont of Paraguaçu.

Since the sectional classification of *Begonia* is morphologically inconsistent and phylogenetically unsatisfactory, and Neotropical species have been poorly sampled and appear to be phylogenetically unresolved, we provide a key to separate them from species that occur in the same surroundings. However, the new species are compared with morphologically similar species and their likely sections (*sensu* Doorenbos et al. 1998) are suggested. Data available for the three new species is still sparse and insufficient to assess them as to their conservation status. Thus, we chose to regard them as Data Deficient (DD; IUCN 2001, 2013) until more information on their ecology and demography is made available.

## Taxonomic treatment

### Begonia delicata

Taxon classificationPlantaeViolalesBegoniaceae

Gregório & J.A.S. Costa sp. nov.

urn:lsid:ipni.org:names:77144526-1

Figures 1 , 2

#### Note.

*Begonia delicata* is similar to *Begonia alchemilloides* A. DC., differing by the presence of a ring of trichomes at the apex of the petiole, stipules and first order bracts with entire margin (vs. laciniate) and staminate flowers with 2 (vs. 4) tepals.

#### Type.

**BRAZIL.** Bahia: São Felipe, Serra da Copioba, 12°50'50"S, 39°05'22"W, Jun 1953 (fl, fr), *G. Pinto 53–55* (holotype: ALCB!).

#### Description.

*Annual herb*, 11–15.5 cm high, monoecious, villous to glabrescent, provided with three types of trichomes, simple, slender trichomes, 1–2.6(–4.5) mm long, trichomes with thickened base, 0.3–0.8 mm long and microscopic, and sparse glandular trichomes. *Stem* 6–8 mm diam., rhizomatous, prostrate, fleshy, pilose, covered by stipules; internodes 1–3 mm long. *Stipules* 0.7–0.75 × 0.3–0.35 cm, ovate, apex long-apiculate, margin entire, with minute hairs to essentially glabrous, carinate, appressed, persistent. *Leaves*: petiole 3.5–9 cm long, cylindrical, villous to glabrescent, ring of trichomes at apex ca. 4 mm long; blade 3.5–7.8 × 4–9.2 cm, reniform, entire, symmetric to slightly asymmetric, basifixed; base cordate; apex rounded; margin crenate, ciliate; sparsely pilose to glabrescent on both surfaces, trichome scars with thickened base, concolorous, light green; venation actinodromous, 7–9 veins at base, membranaceous. *Inflorescence*: dichasial cyme 9–20 cm long, 4–14-flowered; peduncle 7–17,5 cm long, pilose and glandular; first order bracts ca. 1.5 × 0.8 mm, lanceolate, apex apiculate, margin entire, carinate, persistent. *Staminate flowers*: pedicel 9–12 mm long, sparsely glandular to glabrous; tepals 2, white, 6–7 × 5.5–6 mm, ovate to elliptic, apex acute to obtuse, margin entire, glandular on abaxial surface; androecium actinomorphic, stamens 16–22, filaments 0.2–0.4 mm long, free, anthers 1.5–2 mm long, rimose, connective prolonged. *Pistillate flowers*: tepals 5, [only seen in bud]: bracteoles 2, opposite, at base of ovary, lanceolate, persistent [only seen in bud]; styles 3, ca. 0.5 mm long, bifid, branches spirally-arranged, stigmatic papillae covering branches, stigmatic surface papillose, yellow; ovary 7.5–8.2 mm long, trilocular, placentation axile, placenta bifid [obtained from capsules]. *Capsules* ca. 12 × 13 mm [including wings], three-winged, sparsely glandular, dehiscing at the basal portion; wings unequal, larger ones ca. 14 × 5 mm, apex rounded, smaller ones ca. 12 × 3 mm, rounded. *Seeds* ca. 0.2 mm long, elliptic to oblong.

#### Specimen examined

**(paratype). BRAZIL. Bahia:** São Felipe, Serra da Copioba, 12°50'50"S, 39°05'22"W, Oct 1950 (fl), *G. Pinto 587* (RB!).

#### Etymology.

The epithet refers to the fragility and delicacy of the plant.

#### Distribution and habitat.

*Begonia delicata* occurs exclusively in the Recôncavo region (Fig. 2). It is known by only two collections, both from Serra da Copioba, the most recent made in 1953, growing on rocks covered by moss. It has not been found in conservation unit.

**Figure 1.**
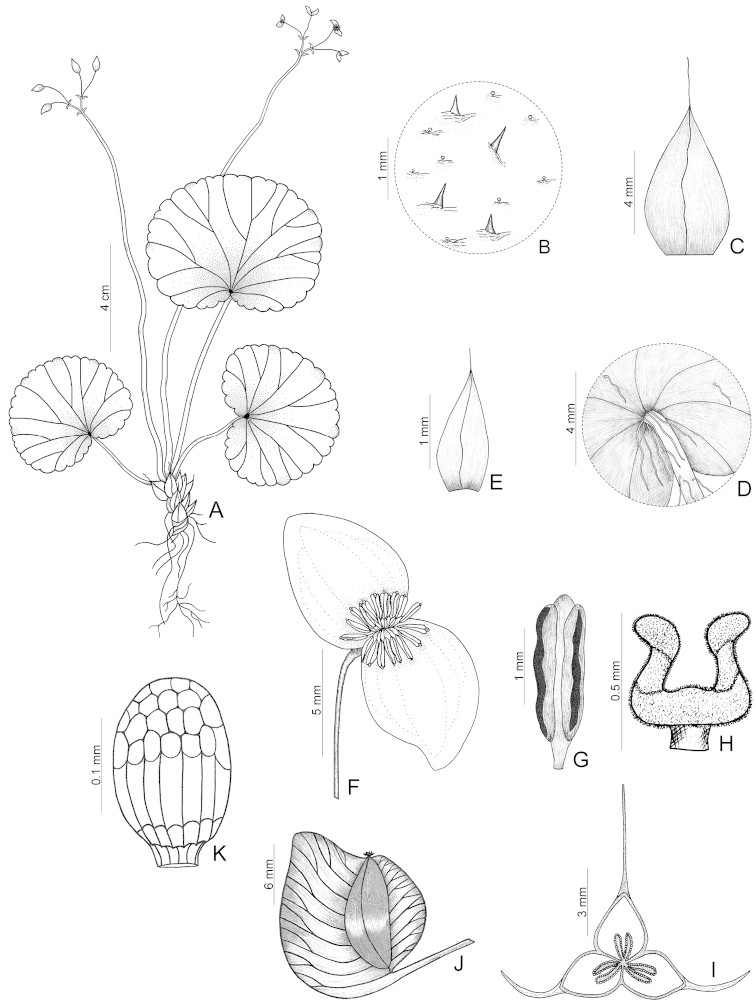
*Begonia delicata*. **A** Habit **B** Detail of indumentum on adaxial surface of leaf-blades **C** Stipule, seen from dorsal side **D** Detail of the ring of trichomes at the apex of the petiole **E** First order bract **F** Staminate flower **G** Stamen **H** Style-branch **I** Ovary, transverse cut, showing placenta **J** Capsule **K** Seed [**A–G** *Pinto 587* (RB); **H–K** holotype *Pinto 53*–*55* (ALCB); drawn by Bernarda Gregório].

**Figure 2.**
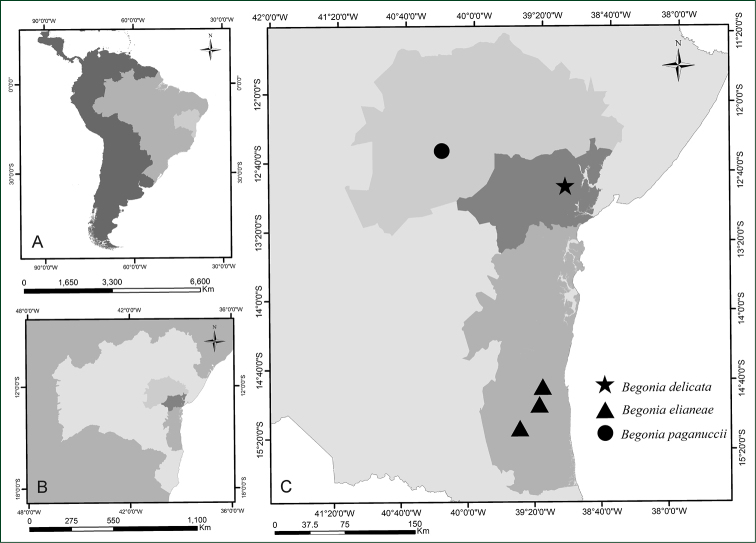
Geographical distribution of three new species of *Begonia*. **A** Latin America showing Brazil and Bahia State **B** Bahia State showing the three political-economic regions of Bahia with new species of *Begonia* **C** Political-economic regions of Bahia showing the occurrence of the three new species.

#### Phenology.

Found flowering in June and October, and with fruits in June.

#### Discussion.

*Begonia delicata* is a small herb easily recognised by the rhizomatous stem covered in stipules, the petioles with a ring of trichomes at the apex, and by the reniform leaf-blades, with crenate margins. Few Brazilian *Begonia* are delicate herbs and, amongst those species, *Begonia alchemilloides* and *Begonia hoehneana* Irmsch. (state of São Paulo) are those that most resemble the new species. *Begonia delicata*, however, can easily be distinguished from both species by the presence of a ring of trichomes at the apex of the petiole and by the staminate flowers with fewer tepals (2 vs. 4). Moreover, the stipules and first order bracts are entire, whereas in *Begonia alchemilloides* they are laciniate, and the leaves are crenate whereas in *Begonia hoehneana* they are dentate. Among the species that occur in Bahia, *Begonia hirtella* Link most closely resembles *Begonia delicata* (see the key below), but can be distinguished by its habit (prostrate in *Begonia delicata* vs. erect in *Begonia hirtella*), the stipules and first order bracts (entire vs. fimbriate), the ring of trichomes at the apex of the petiole (present vs. absent) and the shape of the leaf-blades (reniform vs. ovate). According to the sectional classification of Doorenbos et al. (1998), *Begonia delicata* would belong to the sect. *Doratometra* (Klotzsch) A. DC., which consists of approximately ten annual species, with inconspicuous flowers in relative small inflorescences and two bracteoles below ovary.

### Key to *Begonia* from the Recôncavo region

**Table:** 

1	Stipules with fimbriate margins	
2	Seeds fusiform	***Begonia fischeri* Schrank**
2’	Seeds oblong	***Begonia hirtella* Link**
1’	Stipules with entire margins	
3	Leaf-blades with craspedodromous venation	***Begonia ulmifolia* Willd**
3’	Leaf-blades with actinodromous venation	
4	Stem prostrate; internodes inconspicuous; stipules persistent; ring of trichomes at apex of petiole; leaf-blades with crenate margins; staminate flowers with 2 tepals	***Begonia delicata* Gregório & J.A.S. Costa**
4’	Stem erect; internodes conspicuous; stipules caducous; ring of trichomes absent from apex of petiole; leaf-blades with serrulate margins; staminate flowers with 4 tepals	***Begonia reniformis* Dryand**

### Begonia elianeae

Taxon classificationPlantaeViolalesBegoniaceae

Gregório & J.A.S. Costa sp. nov.

urn:lsid:ipni.org:names:77144527-1

Figures 2 , 3

#### Note.

*Begonia elianeae* is similar to *Begonia besleriifolia* Schott, but is easily distinguished from that species by leaf-blades glabrescent to glabrous on the abaxial surface (vs. sericeous); dichasial cyme with 4 to 8 flowers (vs. 40 to 60); tepals of staminate flowers larger (outer ones: ≥ 15 × 15 mm vs. ≤ 7.5 × 5.3 mm long; inner ones: ≥ 12 × 3 mm vs. ≤ 6 × 3 mm long); filaments shorter (≤ 1.2 mm vs. ≥ 1.5 mm long) and anthers larger (≥ 2 mm vs. ≤ 1.5 mm long); capsules larger (≥ 1.8 cm vs. ≤ 1.5 cm long), with larger wing (≥ 2 cm vs. ≤ 1.2 cm long).

#### Type.

**BRAZIL.** Bahia: Jussari, ca. 9 km North of Jussari, east off of road to Palmira on farm road past cattle farm of Alciato Carvalho, 15°06'58"S, 39°31'58"W, 10 May 1995 (fl, fr), *W.W. Thomas et al. 10863* (holotype: CEPEC!).

#### Description.

*Shrub*, 1–2.5 m high, monoecious, with sparse minute, simple hairs and microscopic glandular hairs to essentially glabrous. *Stem* erect to scandent, fleshy, sparsely pilose, longitudinally striate in herbarium specimens; internodes 1.7–5 cm long. *Stipules* 1.7–3.5 × 0.6–0.8 cm, lanceolate, apex apiculate, margin entire, with minute hairs to essentially glabrous, appressed, persistent. *Leaves*: petiole 1.3–3.3 cm long, cylindrical, with minute hairs to essentially glabrous; blade 13–18.2 × 6.2–8 cm, oblong to obovate, entire, asymmetrical, basifixed; base oblique; apex acuminate; margin entire to slightly undulate, glabrescent to glabrous on both surfaces, discolorous, adaxial surface green, abaxial surface light green to vinaceous; venation craspedodromous, thickened. *Inflorescence*: dichasial cyme 9–15 cm long, 4–8-flowered; peduncle 4,5–6 cm long, with minute hairs to essentially glabrous, vinaceous; first order bracts ca. 15 × 6 mm, obovate, apex rounded, margin entire, caducous. *Staminate flowers*: pedicel 10–14 mm long, glandular; tepals 4, white, the outer pair larger, 15–17 × 15–17 mm, orbicular to ovate, apex rounded, margin entire, concave, glabrescent on abaxial surface, the inner pair 12–14 × 3–4 mm, elliptic to oblanceolate, apex acute to obtuse, margin entire, concave, glabrous; androecium actinomorphic, stamens 26–34, filaments 0.1–1.2 mm long, free, anthers 2–3 mm long, rimose, connective not prolonged. *Pistillate flowers* [only seen in bud]: bracteoles 2, opposite, borne on pedicel, just below the ovary, caducous [not seen; inferred by pedicel scars on flower bud]; pedicel of floral bud ca. 1 cm long; tepals of floral bud 5, 9.2–10 × 5–7 mm, three slightly larger ones, elliptic, apex acute to obtuse, margin entire, glabrescent on the abaxial surface, white [styles damaged]; ovary ca. 7.5 mm long, trilocular, placentation axile, placenta bifid [obtained from capsules]. *Capsules* 1.8–2 × 2.7–3.7 cm [including wings], three-winged, glabrescent, light green, young wings vinaceous, becoming brown at maturity, dehiscing at the basal portion; wings unequal, larger ones 2–2.3 × 1.7–2.1 cm, apex obtuse to rounded, smaller ones 1.5–1.8 × 0.6–0.8 cm, rounded. *Seeds* ca. 0.3 mm long, oblong.

**Figure 3.**
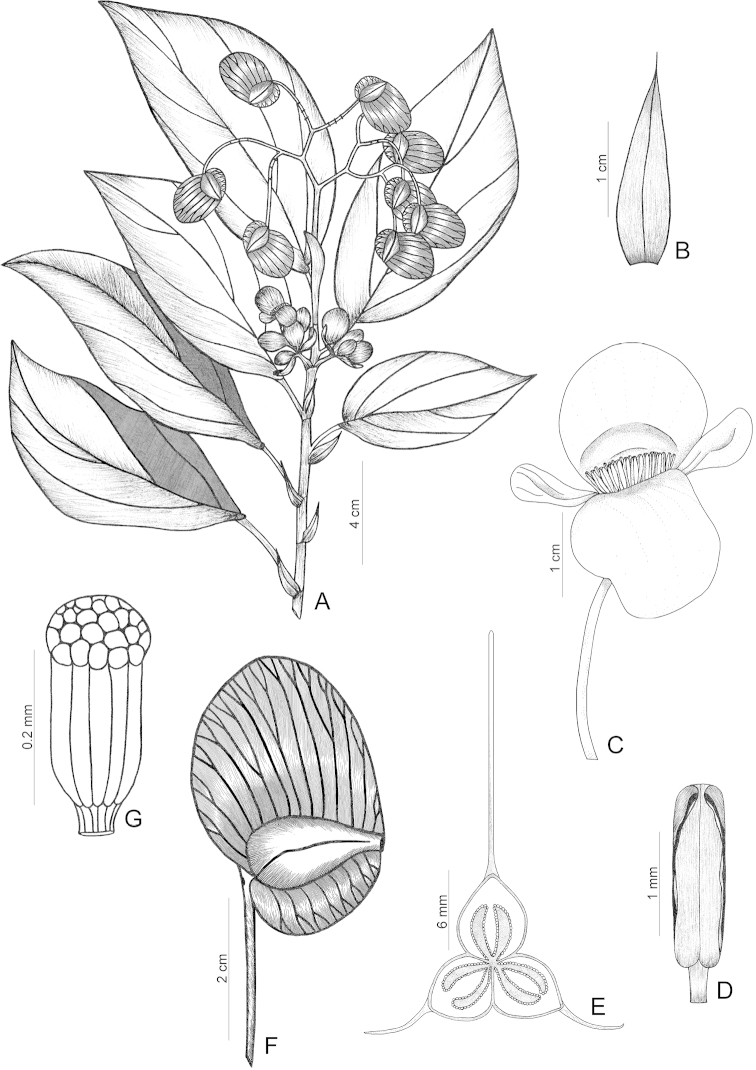
*Begonia elianeae*. **A** Flowering branch **B** Stipule, dorsal side **C** Staminate flower **D** Stamen **E** Ovary, transverse cut, showing placenta **F** Capsule **G** Seed [**A–D** holotype *Thomas 1086* (CEPEC); **E–G** *Fiaschi 1704* (SPF); drawn by Bernarda Gregório].

#### Specimens examined

**(paratypes). BRAZIL. Bahia:** Buerarema, estrada São José da Vitória-Buerarema, ramal à direita, ca. 1 km de São José. Estrada de acesso para Pedra Branca, 15°05'00"S, 39°19'00"W, 15 Oct 2003 (fl, fr), *P. Fiaschi et al. 1704* (CEPEC!; SPF!); Itabuna, fazenda Santa Clara, distrito Ribeirão dos Cachorros, entrada 200 m após a ponte da Bananeira da rodovia BR-101, 12°31'39"S, 40°18'25"W, 21 Aug 1972 (fl), *R.S. Pinheiro 1930* (CEPEC!).

#### Etymology.

The specific epithet is given in honour of Dra. Eliane de Lima Jacques, a botanist who has contributed extensively to our knowledge of *Begonia* from Brazil.

#### Distribution and habitat.

*Begonia elianeae* was found in three localities in southern Bahia, in areas of Atlantic rainforest (Fig. 2), growing on rocks, near pastures or eventually supported by tree trunks, on the edge of trails or disturbed forests. It has not been found in conservation units.

#### Phenology.

Flowering in May, August and October, and with fruits in May and October.

#### Discussion.

*Begonia elianeae* is a shrub characterised by the large oblong to obovate leaf-blades, and by the large, yet few-flowered inflorescences and few-fruited infructescences. In *Begonia*, shrubby species are not so common and only two species with this habit occur in the state of Bahia: *Begonia elianeae* and *Begonia besleriifolia*. Both have craspedodromous leaves and can be distinguished from all other species from southern Bahia with this type of venation using the key below. The most likely section of *Begonia elianeae* is the sect. *Ruizopavonia* A. DC., which consists of suffrutescent plants, with woody stem, straight, craspedodromous leaves, and bracts and styles caducous in fruit.

### Key to the species of *Begonia* from Southern Bahia with craspedodromous leaves

**Table:** 

1	Shrubs	
2	Leaf-blades sericeous on abaxial surface; dichasial cyme with > 40 flowers; staminate flowers: the outer pair of tepals ≤ 7.5× 5.3 mm, the inner pair of tepals ≤ 6 × 3 mm; filaments ≥ 1.5 mm long, anthers ≤ 1.5 mm long; capsules ≤ 1.5 cm long, larger wing ≤ 1.2 cm long	***Begonia besleriifolia* Schott**
2’	Leaf-blades glabrescent to glabrous on abaxial surface; dichasial cyme with 4 to 8 flowers; staminate flowers: the outer pair of tepals ≥ 15× 15 mm, the inner pair of tepals ≥ 12 × 3 mm; filaments ≤ 1.2 mm long, anthers ≥ 2 mm long; capsules ≥ 1.8 cm long, larger wing ≥ 2 cm long	***Begonia elianeae* Gregório & J.A.S. Costa**
1’	Climbers	
3	Stipules lanceolate; leaf-blades with serrate margins; capsules ≤ 1.2 cm wide [including wings], wings equal	***Begonia fruticosa* A. DC.**
3’	Stipules ovate; leaf-blades with entire to sparsely dentate margins; capsules ≥ 1.5 cm wide [including wings], wings unequal, one larger than the others	***Begonia polygonifolia* A. DC.**

### Begonia paganuccii

Taxon classificationPlantaeViolalesBegoniaceae

Gregório & J.A.S. Costa sp. nov.

urn:lsid:ipni.org:names:77144529-1

Figures 2 , 4

#### Note.

*Begonia paganuccii* is similar to *Begonia gardneri* A. DC. However, it can be easily distinguished by the indumentum of dendritic trichomes (vs. simple trichomes); stipules lanceolate and pubescent (vs. ovate and glabrous); staminate flowers with the outer pair ovate to elliptic and the inner pair oblong to oblanceolate (vs. both pairs obovate); endemic to the State of Bahia (vs. endemic to the State of Minas Gerais State).

#### Type.

**BRAZIL.** Bahia: Itaberaba, fazenda Gameleira, entre as fazendas Monte Verde e Leão dos Brejos, 12°24'44"S, 40°32'12"W, 19 Aug 2005 (fl, fr), *L.P. Queiroz et al. 10790* (holotype: HUEFS!; isotypes: CEPEC!, K!, RB!).

#### Description.

*Subshrub*, ca. 3 m high, monoecious, pubescent, with both dendritic greyish trichomes, 0.1–0.4 mm long, and microscopic glandular trichomes. *Stem* erect, fleshy, pubescent; internodes 1–3.5 cm long. *Stipules* 2.5–3 × 0.7–1.5 cm, lanceolate, apex apiculate, margin entire, pubescent, carinate, appressed, caducous. *Leaves*: petiole 6.3–11.6 cm long, cylindrical, pubescent; blade 13–18 × 19–28 cm, transversally elliptic, deeply lobed (lobes approximately half the length of their main vein), 6 or 7 lobes, asymmetric, basifixed; base cordate; lobes with acute apex; margin serrulate; pubescent on both surfaces, more densely so on abaxial surface, discolorous, adaxial surface green, abaxial surface green-cinereous; venation actinodromous, 6 or 7 veins at base, slightly thickened. *Inflorescence*: dichasial cyme 32–39 cm long, ca. 180 flowers; peduncle 23.5–27 cm long, cinereous; first order bracts 4–6 × 1.5–2.5 mm, lanceolate, apex acuminate, margin entire, caducous. *Staminate flowers*: pedicel 1–1.4 cm long, pilose; tepals 4, white, the outer pair larger 6–7.2 × 3–4 mm, ovate to elliptic, apex acute to obtuse, margin entire, concave, glabrescent on abaxial surface, the inner pair 5–6.2 × 1.8–2.3 mm, oblong to oblanceolate, apex obtuse to rounded, margin entire, concave, glabrous; androecium actinomorphic, stamens 32–48, filaments 0.2–0.9 mm long, free, anthers 1–1.3 mm long, rimose, connective prolonged. *Pistillate flowers* [not seen]: bracteoles 2, opposite, borne on pedicel, just below ovary, caducous [scars seen on the pedicel from capsules]; styles 3, 1.6–2 mm long, bifid, branches spirally-arranged, stigmatic papillae covering branches, stigmatic surface papillose, yellow [obtained from capsules]; ovary 5–6.7 mm long, trilocular, placentation axile, placenta entire [observed from capsules]. *Capsules* 6–7.5 × 11–14.6 mm [including wings], three-winged, glabrescent, brown when mature, dehiscing at the basal portion; wings unequal, larger one 5–7 × 6–7 mm, apex obtuse to rounded, smaller ones 5.8–7 × 0.6–1.6 mm. *Seeds* ca. 0.3 mm long, oblong.

**Figure 4.**
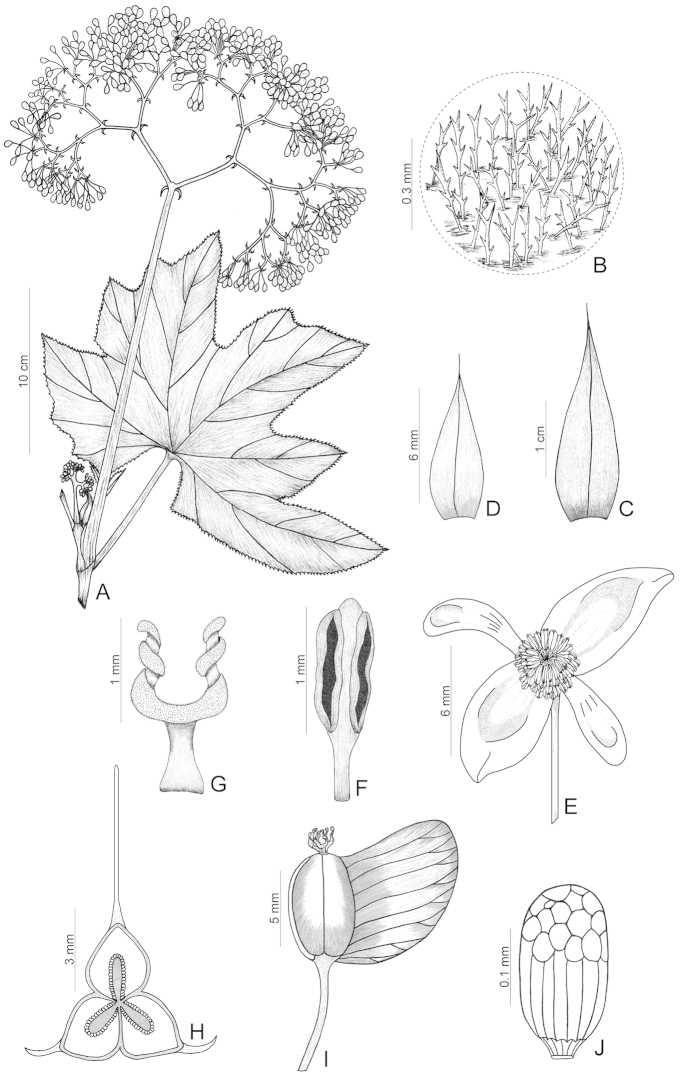
*Begonia paganuccii*. **A** Flowering stem **B** Detail of leaf, showing the dendritic trichomes **C** Stipules, seen from dorsal side **D** First order bract **E** Staminate flower **F** Stamen **G** Style-branch **H** Ovary, transverse cut, showing placenta **I** Capsule **J** Seed [**A–J** holotype *Queiroz 10790* (HUEFS); drawn by Bernarda Gregório].

#### Etymology.

This species is named in honour of Dr. Luciano Paganucci de Queiroz, a great expert on the flora of Bahia, who collected the type material.

#### Distribution and habitat.

*Begonia paganuccii* is known from a single collection from the Área de Relevante Interesse Ecológico (ARIE), a protected area in the municipality of Itaberaba (Fig. 2), region of the Piedmont of Paraguaçu, growing in seasonal forest at 783 m a.s.l. Nevertheless, agriculture and livestock are common around and within the conservation unit.

#### Phenology.

Flowering and fruiting in August.

#### Discussion.

*Begonia paganuccii* is characterised by a dendritic indumentum, stipules lanceolate, and transversally elliptic leaf-blades, 6- or 7-lobed. Trichomes are quite important in the taxonomy of Begoniaceae when combined with other morphological information (Jacques 2002). Some species in Brazil have dendritic trichomes, such as *Begonia egregia* N.E. Br and *Begonia lindmanii* Brade. *Begonia paganuccii* differs from *Begonia egregia* by the basifixed, lobed and transversally elliptic (vs. peltate, entire and ovate to elliptic) leaf-blade, staminate flowers with 4 tepals (vs. 2) and pistillate flowers with trilocular ovary and 3 styles (vs. ovary tetralocular and with 4 styles). It also differs from *Begonia lindmanii* by the lobed (vs. entire) leaf-blade, as well as by the many-flowered dichasial cyme (ca. 180 flowers vs. 10–15 flowers) and pistillate flowers with 2 bracteoles (vs. 3 bracteoles). This species can be distinguished from the remaining species of *Begonia* from the region where it occurs using the key below. Due to the leaves with cystoliths and the entire placenta, it most likely belongs to the sect. *Pritzelia* (Klotzsch) A. DC.

### Key to the species of *Begonia* from Itaberaba, Piedmont Region of the Paraguaçu basin

**Table:** 

1	Leaf-blades with craspedodromous venation	***Begonia ulmifolia* Willd**
1’	Leaf-blades with actinodromous venation.	
2	Stem rhizomatous, prostrate or decumbent; internodes inconspicuous; stipules persistent; leaf-blades with margin entire or slightly undulate	***Begonia pernambucensis* Brade**
2’	Stem not rhizomatous, erect; internodes conspicuous; stipules caducous; leaf-blades with serrulate margin.	
3	Plants covered by microscopic simple and glandular trichomes; stipules triangular; leaf-blades superficially 3–6-lobed, lobes shorter than half the length of their main vein	***Begonia reniformis* Dryand**
3’	Plants covered by microscopic glandular and dendritic trichomes; stipules lanceolate; leaf-blades deeply 6- or 7-lobed, lobes approximately half the length of their main vein	***Begonia paganuccii* Gregório & J.A.S. Costa**

## Supplementary Material

XML Treatment for Begonia delicata

XML Treatment for Begonia elianeae

XML Treatment for Begonia paganuccii
